# The role of LncRNA SNHG3 in human cancers

**DOI:** 10.1007/s12672-025-03640-7

**Published:** 2025-10-03

**Authors:** Jinming Tang, Min Su, Yuhang Xiao, Wei Ou, Wenxiang Wang, Ren-Wang Peng, Zhining Wu

**Affiliations:** 1 Hunan Clinical Medical Research Center of Accurate Diagnosis and Treatment for Esophageal Carcinoma, The Affiliated Cancer Hospital of Xiangya School of Medicine, Central South University/Hunan Cancer Hospital, Changsha, 410013 Hunan China; 2 Thoracic Surgery Department 2, The Affiliated Cancer Hospital of Xiangya School of Medicine, Central South University/Hunan Cancer Hospital, Changsha, 410013 Hunan China; 3 Hunan RunKun Pharmaceutical Co., Ltd., Yueyang, 414003 Hunan China; 4 Department of Pharmacy, Xiangya Hospital of Xiangya School of Medicine, Central South University, Changsha, 410013 Hunan China; 5 Department of Pharmacy, YUEYANG Central Hospital, Yue Yang, 414003 Hunan China; 6 Department of General Thoracic Surgery, Inselspital, Bern University Hospital, Murtenstrasse 50, 3008 Bern, Switzerland; 7 Department of BioMedical Research, University of Bern, Murtenstrasse 50, 3008 Bern, Switzerland

**Keywords:** Long noncoding RNA, SNHG3, Human cancer, Oncogene

## Abstract

An essential category of non-protein-coding RNA molecules, known as long noncoding RNAs (lncRNAs), are naturally occurring RNA sequences exceeding 200 nucleotides in length. Accumulating evidence has unveiled the significant involvement of lncRNAs in various cancers, where they are known to be a key factor for tumors’ statement changing, e.g., modulating proliferative, apoptotic, migratory and chemotherapy resistance in cells. In addition, lncRNAs are also regarded as potential diagnostic and prognostic tumor biomarkers. Studies have suggested that lncRNAs modulate biological processes by regulating linear RNA transcription and protein production. A recently discovered long non-coding RNA, SNHG3, is identified to have associations with cancer, which displays atypical expression patterns and functions as an oncogene in various cancers. Additionally, SNHG3 has been demonstrated to hold promise as a diagnostic biomarker and a tool for prognostic assessment in cancer patients. This review summarizes current knowledge about SNHG3’s expression, functional roles, molecular mechanisms, and implications for diagnosis and prognosis in human cancers.

## Introduction

Global cancer prevalence poses a substantial challenge to public health, ranking second among worldwide mortality causes [1]. Despite advancements, numerous patients with advanced cancer do not respond effectively to existing treatments [2]. The need for innovative therapeutic targets drives ongoing research. Long non-coding RNAs (lncRNAs) are RNA molecules over 200 nucleotides long, and do not exhibit strong conservation [3]. Although lncRNAs possess minimal protein-coding potential, they can play pivotal roles in gene expression regulation, influencing a range of biological processes.

LncRNA small nucleolar RNA host gene 3 (SNHG3, GenBank accession no. NR_034119), also known as U17 Host Gene (U17HG), is located at 1q35.3 [4]. SNHG3 consists of four exons spanning a total length of 4950 bp (chr1: 28,505,942− 28,510,892). It was discovered in 1998, and was reported to play a role in dysregulating translation and ribosome biogenesis during Alzheimer’s disease progression [4, 5]. SNHG3 exhibits higher expression levels in the brain, adrenal and testes compared to other human tissues, according to RNA-seq data. Recent evidence reveals SNHG3 plays a critical role in the occurrence and development of many malignant tumors. In the current review, we summarized recently reported findings about the expression, biological functions, related clinical features, underlying mechanisms and clinical applications of SNHG3. This will help to clarify SNHG3’s crucial role and offer guidance for SNHG3 studies in the future.

## SNHG3 expression in malignancies

SNHG3 is generally upregulated in multiple malignancies, including bladder cancer [6, 7], breast cancer [8–11], clear cell renal cell carcinoma [12, 13], colorectal cancer [14, 15], esophageal carcinoma [16], gastric cancer [17–19], glioma [20, 21], hepatocellular carcinoma [22], laryngeal squamous cell carcinoma [23], lung cancer [24, 25], osteosarcoma [26], ovarian [27–29], and prostate cancer [30–32] (Table 1). The corresponding cancer cells also tend to show increased SNHG3 amounts compared to normal cells.

Research using the Cancer Genome Atlas Prostate Adenocarcinoma (TCGA-PRAD) database revealed higher SNHG3 expression in prostate cancer patients with bone metastasis (PC/BM) compared to non-metastatic cases (PC/nBM) [32]. SNHG3 was also found at increased levels in prostate cancer tissues versus adjacent normal tissues (*n* = 26), indicating a potential association between SNHG3 and prostate cancer development, and further increased in PC/BM cases (*n* = 25) compared to PC/nBM cases (*n* = 35) from in-house clinical samples. Moreover, a comparison between metastatic prostate cancer tissues obtained from bone (B-PC, *n* = 21) and primary prostate cancer tissues (P-PC, *n* = 60) showed increased SNHG3 expression in B-PC samples. These findings suggest that SNHG3 expression levels may be indicative of the progression and metastasis of prostate cancer.

However, in papillary thyroid carcinoma, there are contrary reports of SNHG3 expression [33]. Initial investigations into SNHG3 expression using the GEPIA platform and GEO datasets revealed reduced expression in papillary thyroid carcinoma tissues when being contrasted to normal thyroid tissues. The results were further corroborated by RT-PCR analysis, which demonstrated a more than 1.5-fold decrease in SNHG3 expression in papillary thyroid carcinoma tissues (38/62, 61.3%) relative to matched normal thyroid tissues. Consistent with these observations, SNHG3 expression levels were reduced in three papillary thyroid carcinoma cell lines in comparison to normal thyroid follicular cells. Notably, low SNHG3 expression was significantly associated with worse clinical outcomes for papillary thyroid carcinoma patients, particularly in terms of recurrence free survival (RFS). The authors did not analyze the discrepancies in the expression and role of SNHG3 between papillary thyroid carcinoma and various other cancers. The variations among cancers might lack adequate explanations, and the discrepancy needs to be further explored.

**Table 1:** Expression of SNHG3 in various cancers

Cancer type	Expression in tissue	sample size	Expression in cancer cells	Relative normal cell lines	Cancer cell lines	Functional role	References
Bladder cancer	Up	70	Up	SV-HUC1	5637, T24	Promote proliferation, migration, invasion, EMT	[6]
	Up	58	Up	SV-HUC-1	J82, 5637, T24, SW780, UM-UC-3	Promote proliferation, invasion, migration, angiogenesis	[7]
Breast Cancer	Up	60	Up	MCF-10 A	MCF-7, MDA-MB-231, HCC1937, BT474, SKBr-3	Promote proliferation, migration, invasion, EMT	[8]
	Up	30	Up	MCF-10 A	MDA-MB-231, BT-549, MDA-MB-468	Promote proliferation, migration, invasion	[9]
	Up	40	Up	MCF-10 A	T47D, MDA-MB-231, MDA-MB-468, MCF7, BT-549, SK-BR-3	Promote proliferation, migration, invasion	[10]
	-	-	Up	MCF-10 A	MCF-7, MDA-MB-231	Promote proliferation, migration, invasion, EMT	[11]
Clear cell renal cell carcinoma	Up	70	Up	HK2	A498 and 786-O	Promote migration, invasion	[12]
	Up	62	-	-	-	-	[13]
Colorectal cancer	Up	50	Up	NCM460	SW480, SW620, HCT8, HT29	Promote proliferation, invasion	[14]
	Up	40	Up	NCM460	HT29, HCT116, SW480, LoVo	Promote proliferation	[15]
esophageal cancer	Up	348		-	KYSE-150 and Eca-9706	Promote proliferation, inhibit apoptosis	[16]
Gastric Cancer	Up	25	Up	GES-1	HGC-27, MGC-803	Promote proliferation, metastasis	[17]
	Up	26	Up	GES-1	BGC-823, AGS, MGC-803, HGC-27	Promote proliferation, migration, invasion	[18]
	Up	60	Up	GES-1	MGC-803, AGS, BGC-823, SGC-7901, MKN-45, GC-27	Promote proliferation, migration, invasion, EMT	[19]
Glioma	Up	42	Up	NHA	A172, SHG44	Promote migration, invasion, inhibit apoptosis	[20]
	Up	16	−	−	U-87-MG and U-251-MG	Promote proliferation, migration, invasion	[21]
Hepatocellular carcinoma	Up	47	Up	L02	HepG2, HCCLM3	Promote proliferation, migration, EMT, inhibit apoptosis	[22]
Laryngeal squamous cell carcinoma	Up	25	Up	NP69	TU177, AMC-HN-8	Promote viability, glycolysis, inhibit apoptosis	[23]
Lung Cancer	Up	15	Up	BEAS-2B	A549, HCC827, H2170, H520	Promote proliferation, migration, invasion, EMT	[24]
Osteosarcoma	Up	54	Up	hFOB 1.19	Saos2, MG63, U2OS, HOS	Promote migration, invasion	[26]
Ovarian Cancer	Up	40	Up	HOSE	A2780, SKOV3, OVCAR3, OV90,	Promote proliferation, migration	[27]
	Up	76	Up	HOSE	SKOV3, OVCAR3, A2780, ES2	Promote proliferation, invasion	[28]
	Up	96	Up	IOSE	SKOV3, HeyA8, A2780	Promote proliferation, migration, invasion	[28]
Papillary Thyroid Carcinoma	Down	62	Down	Nthy-ori 3 − 1	BCPAP, KTC-1 and TPC-1	Inhibit proliferation, migration	[33]
Prostate cancer	Up	30	Up	RWPE-1	PC-3, DU145, VCaP, LNCaP	Promote proliferation, migration, invasion	[30]
	-	-	Up	RWPE-1	PC3, DU145, 22RV1, LNCaP	Promote proliferation, migration, EMT, apoptosis.	[34]
	Up	40	Up	WPMY-1	PC-3, Du 145, LNCaP, 22RV1	Promote proliferation, migration, invasion, inhibit apoptosis	[31]
	Up	26	Up	RWPE-1	DU145, VCaP, LNCaP, C4-2B, 22RV1, PC3	Promote proliferation, migration, invasion	[32]

## Regulation of SNHGin cancer

Studies have shown that SNHG3 expression is regulated by genetic and epigenetic mechanisms, as well as antitumor drugs. However, the upstream regulatory mechanisms of SNHG3 remain far from being fully elucidated. To determine the factor responsible for increased SNHG3 expression in breast cancer, potential binding factors for the SNHG3 promoter region were predicted using JASPAR tools [10]. STAT3 showed the strongest binding potential among the predicted factors. In vitro experiments revealed that SNHG3 expression significantly decreased when STAT3 was knocked down and increased when STAT3 was overexpressed in breast cancer cells. Chromatin immunoprecipitation experiments confirmed that STAT3 directly interacts with the SNHG3 promoter region, and luciferase reporter assays validated the binding sequence. The results collectively demonstrate that STAT3 is essential to the regulating SNHG3 expression in breast cancer cells.

A study [13] discovered that the methylation levels of two CpG sites, cg07807470 (*r* = − 0.5662) and cg15161854 ( *r*= − 0.6244), were inversely related to SNHG3 expression. In 15 clear cell renal cell carcinoma samples, cg07807470 and cg15161854 methylation levels were notably lower than in adjacent normal tissues. To further investigate the connection between methylation levels and SNHG3 expression, cg15161854 levels were tested using pyrosequencing in an additional 36 clear cell renal cell carcinoma samples. Although these findings suggest that DNA methylation may regulate SNHG3 expression, more research is necessary to fully understand the mechanisms involved.

Platinum-based drugs combined with 5-fluorouracil are commonly used as the primary treatment for esophageal cancer, but platinum resistance often results in local recurrence and poor patient outcomes [35]. A study [16] found that SNHG3 expression is increased in esophageal cancer cells following treatment with platinum drugs such as cisplatin, carboplatin, and oxaliplatin. However, the exact mechanisms by which platinum drugs regulate SNHG3 expression remain unclear.

## SNHG3 functions in malignancies

The research has manifested that numerous factors play a role in the onset and advancement of cancer, with key aspects being resistance to cell death, activation of pathways that lead to invasion and metastasis, and increased resistance to chemotherapy [36]. Recent studies have highlighted the crucial role of SNHG3 in regulating oncogenes and tumor suppressors, ultimately influencing the key characteristics of cancer cells (Table 1).

### SNHG3 in cell viability and proliferation

In vitro gain- or loss- of function experiments have shown that SNHG3 plays a crucial role in cancer cell proliferation. SNHG3 was found to promote cell proliferation by facilitating cell cycle progress. In prostate cancer cells, decreasing SNHG3 expression reduced the number of cells in the S phase, while increasing SNHG3 expression accelerated cell cycle transition from the G0/G1 phase to the S phase [31]. In ovarian cancer cells, inhibiting SNHG3 led to G1/G0 cell cycle arrest, suppressing malignant characteristics [29]. In lung adenocarcinoma (LUAD) cells, increased SNHG3 expression led to a decrease in cells in the G1 phase and an increase in the S phase [37]. On the other hand, decreasing SNHG3 expression reduced the S phase cell ratio and lowered levels of cell cycle regulatory proteins (CDK6, CDK4, and Cyclin D1) [10]. Additionally, animal models with tumors showed that reducing SNHG3 led to smaller tumor volume and weight, including bladder cancer [6, 7], breast cancer [8, 10, 11], colorectal cancer [15], gastric cancer [18, 19], glioma [21], laryngeal squamous cell carcinoma [23], ovarian cancer [29], papillary thyroid carcinoma [33], and prostate cancer [30] (Table 2). High SNHG3 expression is associated with larger tumors in various cancers, such as bladder cancer [6].

Cancer-related exosomes contribute to cancer progression [38]. SNHG3 expression is abnormally elevated in cancer-associated fibroblasts (CAFs) from breast cancer patients compared to normal breast cells [39]. CAF-secreted exosomes increased breast cancer cell growth in a concentration-dependent manner. Exosomes from CAFs with reduced SNHG3 expression suppressed glycolysis metabolism and cell proliferation in breast cancer cells, while exosomes from CAFs with increased SNHG3 expression enhanced glycolysis metabolism and cell proliferation. In colorectal cancer, SNHG3 expression levels in CAFs and CAF-derived extracellular vesicles (EVs) were higher than in normal fibroblasts (NFs) and NF-EVs. CAF-EVs facilitated colorectal cancer cell proliferation by transferring SNHG3 into colorectal cancer cells, increasing SNHG3 expression [40].

**Table 2:** In vivo functional characterization of SNHG3 in cancer

Cancer type	Cancer cell lines	Animal	Role in tumor growth	Role in tumor metastasis	References
Bladder cancer	5637	BALB/c nude mice	Promote	–	[6]
	T24	NOD-PrkdcscidIl2rgem1/Smoc mice	Promote	–	[7]
Breast cancer	MCF-7	nude mice	Promote	–	[11]
	MDA-MB-231	BALB/c nude mice	Promote	Promote liver metastasis	[10]
	MCF-7	BALB/c nude mice	Promote	–	[8]
Colorectal cancer	SW480	BALB/c nude mice	Promote	–	[15]
Gastric cancer	HGC-27	BALB/c nude mice	Promote	–	[18]
	SGC-7901	BALB/c mice mice	Promote	Promote lung metastasis	[19]
Glioma	U-87-MG, U-251-MG	BALB/c nude mice	Promote	–	[21]
Laryngeal squamous cell carcinoma	AMC-HN-8	BALB/c nude mice	Promote	–	[23]
Ovarian Cancer	HeyA8	BALB/c nude mice	Promote	–	[29]
Papillary Thyroid Carcinoma	BCPAP	BALB/c nude mice	Promote	–	[33]
Prostate cancer	PC-3	BALB/c nude mice	–	–	[30]
	Du 145	BALB/c nude mice	Promote	–	[31]

### SNHG3 in cell death

Apoptosis, autophagic, and necrotic cell deaths are key mechanisms for cells to die [41]. SNHG3 is known to block apoptosis in various cancers like gastric cancer [19], glioma [20], hepatocellular carcinoma [22], laryngeal squamous cell carcinoma [23], lung cancer [25], and prostate cancer [31, 34]. This function might be linked to controlling apoptotic proteins. In SNHG3-silenced lung cancer cells, proteins promoting cell death (Bax, cleaved caspase-3, cleaved caspase-9) increased.

### SNHG3 in cancer metastasis

Metastasis is a common cause of cancer deaths [42, 43]. Studies show that SNHG3 regulates migration and invasion pathways in cancer cells. It impacts metastasis, mainly by influencing epithelial-to-mesenchymal transition (EMT), where cells change to acquire invasive properties. Reducing SNHG3 inhibits EMT and modifies EMT-related proteins (increases E-cadherin, decreases N-cadherin and vimentin) [6, 8, 11, 19, 22, 24, 34]. In vivo studies demonstrate silencing SNHG3 reduces breast cancer liver metastasis [11] and gastric cancer lung metastasis [19]. SNHG3 is linked to lymph node/distant metastases and Tumor Node Metastasis (TNM) stage in cancers like bladder [6, 7], breast [10], colorectal [14], gastric [25], liver [22], ovarian [19, 28, 29], and papillary thyroid carcinoma [33].

### SNHG3 in cancer angiogenesis

Angiogenesis is of essential importance in tumor formation, invasion, and metastasis, making it crucial for tumor progression. In vitro experiments show that reducing SNHG3 expression weakens bladder cancer cells’ ability to promote tube formation, while increasing SNHG3 promotes angiogenesis [7]. This suggests that SNHG3 may be critical in regulating angiogenesis in cancer.

## Mechanisms underlying SNHG3’s effects in malignancies

**Fig. 1:**
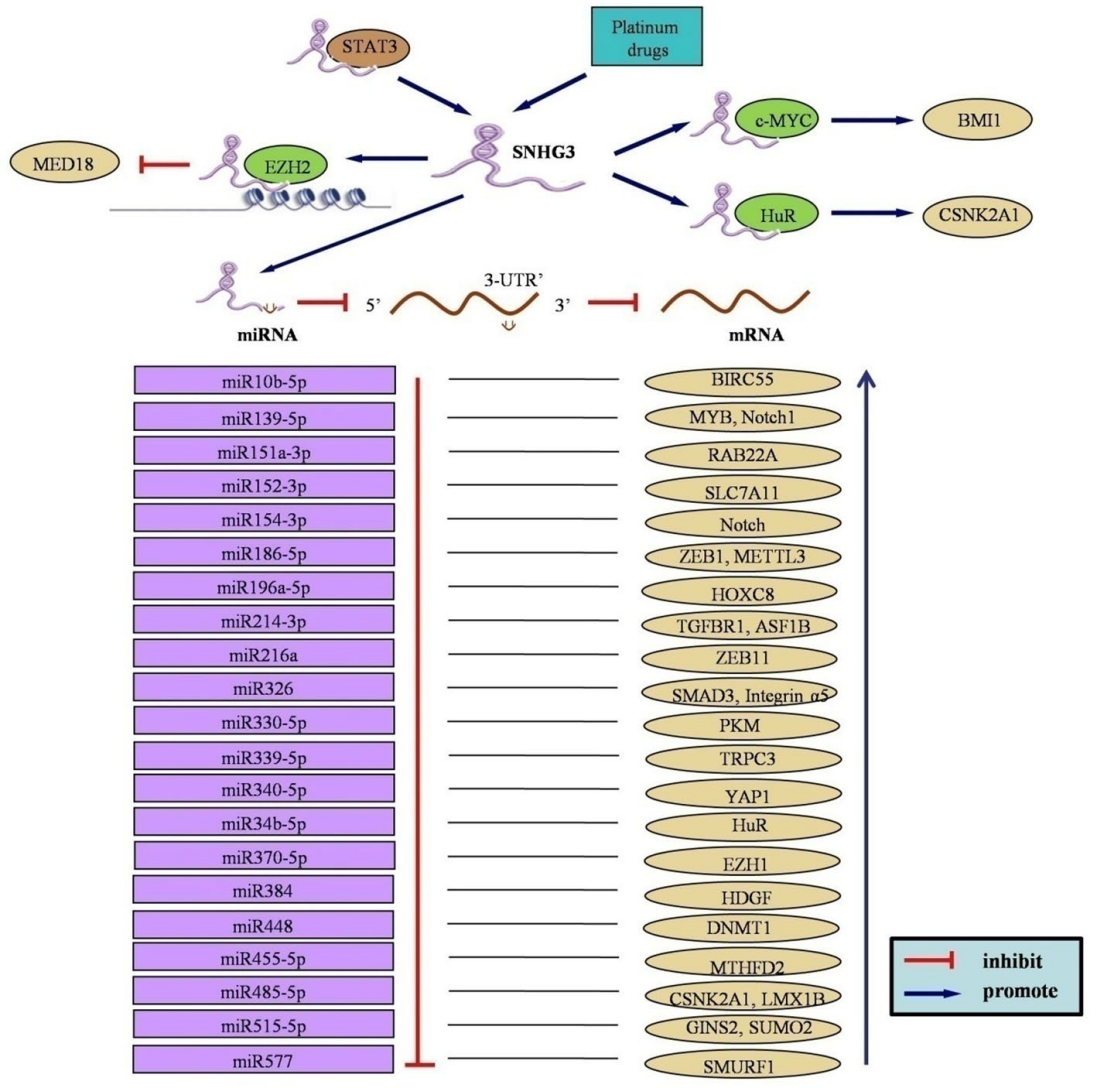
Upstream regulatory and downstream molecular mechanisms underlying SNHG3 in human cancers

**Fig. 2:**
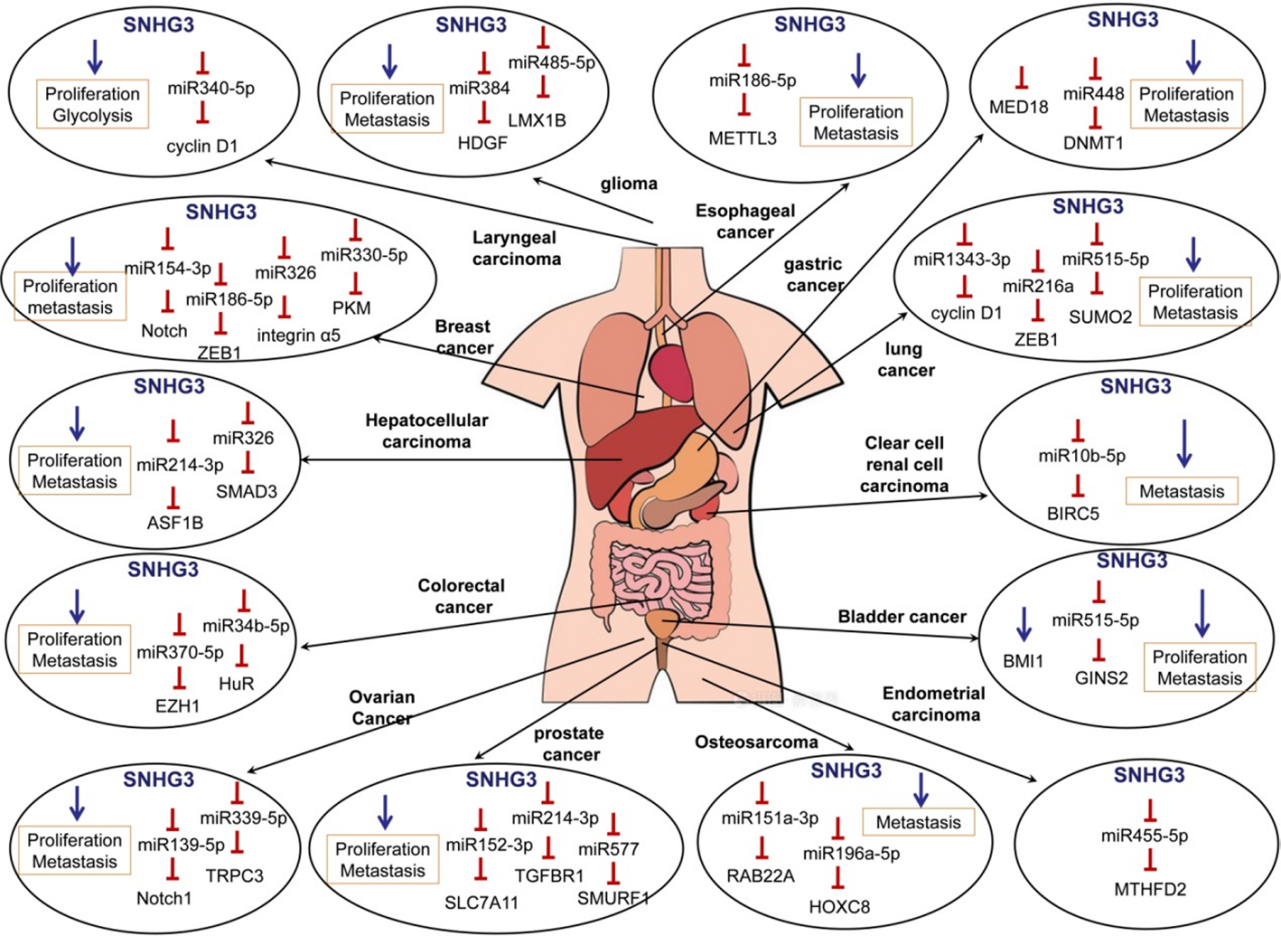
The regulatory mechanisms and functional roles of SNHG3 in multiple human cancers

Studies show that some lncRNAs regulate cell processes by affecting epigenetic changes, influencing transcription and splicing, interacting with RNA-binding proteins, and working as miRNA sponges [44]. SNHG3’s localization in both the cytoplasm and nucleus enables it to perform significant regulatory functions during transcription and post-transcriptional processes (Fig. 1). Although existing studies have partially elucidated SNHG3’s regulatory mechanisms in various tumors, most research has focused on its role as a competing endogenous RNAs (ceRNA), and there is a lack of studies on its broader molecular mechanisms, particularly scaffolding effects, remain underexplored (Fig. 2). Future research such as integrating bioinformatics tools could systematically investigate SNHG3’s potential mechanisms in oncogenesis. Elucidating the regulatory mechanisms would not only advance fundamental understanding of SNHG3 but also facilitate its translational applications in cancer diagnostics and targeted therapeutics.

### SNHG3 serves as a CeRNA

Recent research on the ceRNA hypothesis has significantly contributed to the field of RNA biology by uncovering a novel RNA interaction mechanism. Studies have shown that SNHG3 functions as a ceRNA, regulating gene expression by acting as a molecular sponge for miRNAs, including miR10b-5p/BIRC5 [12], miR139-5p/MYB [18], miR139-5p/Notch1 [27], miR151a-3p/RAB22A [26], miR152-3p/SLC7A11 [31], miR154-3p/Notch [8], miR186-5p/ZEB1 [11], miR186-5p/METTL3 [16], miR196a-5p/HOXC8 [45], miR214-3p/TGFBR1 [32], miR214-3p/ASF1B [46], miR216a/ZEB1 [25], miR326/SMAD3 [22], miR326/integrin α5 [9], miR330-5p/PKM [39], miR339-5p/TRPC3 [29], miR340-5p/YAP1 [23], miR34b-5p/HuR [40], miR370-5p/EZH1 [14], miR384/HDGF [20], miR448/DNMT1 [17], miR455-5p/ MTHFD2 [47], miR485-5p/ LMX1B [21], miR485-5p/CSNK2A1 [10], miR515-5p/GINS2 [6], miR515-5p/ SUMO2 [24], miR577/SMURF1 [34], and so on (Table 3). Various experiments, such as luciferase reporter, RNA immunoprecipitation (RIP) and RNA pull-down assays, have been conducted to find miRNA-binding sites on SNHG3. Additionally, functional assays demonstrated that miRNA and its target mRNA control SNHG3’s functions.

**Table 3:** CeRNA function of SNHG3 in cancer

Cancer type	SNHG3 target miRNA	Validated method	miRNA target gene	References
Bladder cancer	miR515-5p	Luciferase reporter assay	GINS2	[6]
Breast cancer	miR154-3p	Luciferase reporter assay, RNA pull down	Notch	[8]
	miR186-5p	Luciferase reporter assay	ZEB1	[11]
	miR326	Luciferase reporter assay	integrin α5	[9]
	miR330-5p	Luciferase reporter assay	PKM	[39]
	miR485-5p	Luciferase reporter assay, pull-down, RIP	CSNK2A1	[10]
Clear cell renal cell carcinoma	miR10b-5p	Luciferase reporter assay	BIRC5	[12]
Colorectal cancer	miR34b-5p	Luciferase reporter assay, RNA pull down	HuR	[40]
	miR370-5p	Luciferase reporter assay	EZH1	[14]
Endometrial Carcinoma	miR455-5p	Luciferase reporter assay	MTHFD2	[47]
Esophageal cancer	miR186-5p	Luciferase reporter assay, RNA pull down	METTL3	[16]
Gastric cancer	miR139-5p	Luciferase reporter assay, RIP	MYB	[18]
	miR448	Luciferase reporter assay	DNMT1	[17]
Glioma	miR384	Luciferase reporter assay	HDGF	[20]
	miR485-5p	Luciferase reporter assay	LMX1B	[21]
Hepatocellular carcinoma	miR214-3p	Luciferase reporter assay	ASF1B	[46]
	miR326	Luciferase reporter assay	SMAD3	[22]
Laryngeal squamous cell carcinoma	miR340-5p	Luciferase reporter assay	YAP1	[23]
Lung Cancer	miR216a	Luciferase reporter assay	ZEB1	[25]
	miR515-5p	Luciferase reporter assay	SUMO2	[24]
Osteosarcoma	miR151a-3p	Luciferase reporter assay, RNA pull down, RIP	RAB22A	[26]
	miR196a-5p	Luciferase reporter assay	HOXC8	[45]
Ovarian Cancer	miR139-5p	Luciferase reporter assay	Notch1	[27]
	miR339-5p	Luciferase reporter assay, RNA pull down, RIP	TRPC3	[29]
Prostate cancer	miR152-3p	Luciferase reporter assay, RIP	SLC7A11	[31]
	miR214-3p	Luciferase reporter assay	TGFBR1	[32]
	miR577	Luciferase reporter assay, RNA pull down	SMURF1	[34]

### SNHG3 interacts with RNA binding proteins

RNA-binding proteins (RBPs) interact with mRNA regions, controlling their stability and translation. Some lncRNAs recruit RBPs, affecting downstream mRNA molecules [48]. In bladder cancer cells, decreased SNHG3 downregulates expression of *MYC* genes [7]. In vitro assays show c-MYC protein binds SNHG3. Reducing SNHG3 or c-MYC decreases B lymphoma Mo-MLV insertion region 1 (*BMI1)* mRNA stability. Interestingly, overexpressing SNHG3 leads to increased stability of *BMI1* mRNA through its interaction with c-MYC. This indicates a dynamic interplay between SNHG3 and c-MYC in modulating the stability of *BMI1* mRNA. I RIP assays have uncovered EZH2’s association with SNHG3 [19]. It has been confirmed that EZH2 binds directly to the *MED18* promoter region. Further studies show that reduced SNHG3 weakens this interaction, while SNHG3 overexpression enhances it. SNHG3 and EZH2 can simultaneously bind to the *MED18* promoter, leading to increased methylation levels and epigenetic regulation of *MED18* transcription. In breast cancer cells, studies have revealed that HuR protein directly binds to SNHG3 [10]. Upon knocking down HuR, a notable decrease in CSNK2A1 expression is observed. This decrease counters the upregulation of CSNK2A1 induced by SNHG3 overexpression. These findings suggest that SNHG3 cooperates with HuR to control CSNK2A1 expression, potentially impacting cellular pathways.

## SNHG3 as a cancer biomarker

Recent findings suggest that abnormal SNHG3 expression could be a diagnostic and prognostic factor for various cancers. Elevated SNHG3 levels were significantly associated with poor OS in bladder cancer [6], clear cell renal cell carcinoma [12, 13], colorectal carcinoma [14, 15], gastric cancer [18, 25, 49], osteosarcoma [26], and ovarian cancer [19, 28, 29, 32]. Additionally, increased SNHG3 was associated with poor progression-free survival (PFS) in bladder cancer [6], metastasis-free survival (MFS) [19] and bone metastasis-free survival (BMFS) [32] in ovarian cancer. Apart from survival data, SNHG3 expression was found to correlate with various clinical features. High SNHG3 levels in tumor tissues showed marked associations with clinicopathological characteristics like tumor size, tumor stage, FIGO stage, histological grade, lymph node metastasis, and TNM stage (Table 4).

**Table 4:** Involvement of SNHG3 in cancer prognosis

Cancer type	Prognostic indicator	Associated clinical features	References
Bladder cancer	OS, DFS	Tumor size, metastasis	[6]
	-	Tumor grade, muscle invasion, TNM stage	[7]
	-	Tumor stage, lymph node metastasis	[10]
Clear cell renal cell carcinoma	OS		[12]
	OS	Tumor stage	[13]
Colorectal arcinoma	OS	Lymph node metastasis	[14]
	OS	-	[15]
Gastric cancer	OS	Tumor stage	[18]
Glioma	–	Tumor grade	[21]
Hepatocellular carcinoma	–	TNM stage	[22]
Osteosarcoma	OS		[26]
Ovarian cancer	OS	FIGO stage, lymph node metastasis	[28]
	OS	Histological grade, lymph node metastasis	[29]
	OS, MFS	Lymph node metastasis	[19]
	OS, BMFS		[32]
Papillary Thyroid Carcinoma	–	TNM stage	[33]

In prostate cancer [32], SNHG3 might serve as a diagnostic biomarker, as its expression levels can successfully differentiate prostate cancer tissues from normal prostate tissues based on the TCGA-PRAD database (area under the curve (AUC) = 0.917, 95% confidence interval [CI]: 0.867–0.966). Multivariate Cox regression analysis revealed that elevated SNHG3 expression could serve as an independent predictor of mortality (hazard ratio [HR] = 1.752, 95% CI: 1.164–2.657, *p* = 0.008) and disease progression (HR = 7.598, 95% CI: 0.944–61.152, *p* = 0.037) in prostate cancer. Moreover, high SNHG3 levels were also found to be associated with poor overall survival (OS) (HR = 8.74, 95% CI: 2.53–30.19, *p* = 0.008), and PFS (HR = 1.88, 95% CI: 1.25–2.83, *p* = 0.002) based on the database. Furthermore, increased SNHG3 levels correlated with shorter OS (HR = 3.02, 95% CI: 1.16–7.86, *p* = 0.028) and BMFS (HR = 2.41, 95% CI: 1.03–5.61, *p* = 0.04) in prostate cancer patients, as demonstrated by in-house tissue samples.

In breast cancer, eight lncRNAs were identified from TCGA data for diagnosing breast cancer, with the screening criteria AUC value > 0.7 [50]. Among these, SNHG3 showed a 0.706789 AUC value. SNHG3 also had good diagnostic ability in tissue samples based on the GEO GSE134359 dataset (AUC = 0.884009). However, the AUC value of the SNHG3 was all less than 70% in the extracellular vesicle dataset exoRBase GSE93078 (AUC = 0.440285), suggesting that SNHG3 lacks strong diagnostic potential in extracellular vesicles for breast cancer.

Several studies combined SNHG3 with other lncRNAs to investigate its diagnostic and prognostic value further. However, most of the models were developed using bioinformatics approaches, further large prospective clinical cohorts are needed to verify the clinical utility of SNHG3. Moreover, to establish SNHG3 as a reliable diagnostic or prognostic tool, further research is needed to evaluate its expression, sensitivity, and stability in non-invasive body fluids.

A prognostic model using four lipid metabolism-related lncRNAs was developed from the TCGA-LUAD dataset [51]. Risk scores were calculated using the equation: SNHG3 × 0.752442869 + LINC00857 × 1.469417445 + EP300-AS1 × 0.757747569 + TBX5-AS1 × 0.660575856. In the study, patients with high-risk scores (*n* = 175) had worse OS compared to those with low-risk scores (*n* = 175). The accuracy of survival prediction at 1, 3, and 5 years was 0.639, 0.631, and 0.626, respectively. In the validation group from the GSE50081 dataset, high-risk patients (*n* = 90) had worse outcomes than low-risk patients (*n* = 91). The accuracy of survival prediction at 1, 3, and 5 years was 0.724, 0.666, and 0.651, respectively. All four prognostic lncRNAs showed good diagnostic ability in the TCGA and GSE31210 datasets, with accuracy exceeding 0.78, whereas the overall diagnostic model using the four prognostic lncRNAs proved more effective than methods using a single gene, with AUCs of 0.997 in TCGA-LUAD cohort and 0.939 in GSE31210 dataset.

To develop an oxidative phosphorylation-related gene signature, a seven-gene signature was established using LASSO Cox analysis in the TCGA-LUAD training dataset (522 samples) [52]. The prognostic signature was established used 366 samples (70%) from the training set, based on the risk score formula: SNHG3 × (− 0.1752) + CFTR × (-0.0704) + MAP1LC3C × (-0.0229) + LDHA × 0.3428 + HSPD1 × 0.2827 + TWIST1 × 0.0126 + COX6B2 × 0.0734. Survival analysis showed that patients with high-risk scores had significantly worse OS than those with low-risk scores (HR = 3.4; 95% CI = 2.3–4.9; *p* < 0.001). The accuracy of survival prediction at 3 and 5 years was 0.703 and 0.651, respectively. In the 30% test group, the survival rates of the two patient groups significantly differed (HR = 2.6; 95% CI = 1.5–4.5; *p* < 0.004), with AUCs of 0.632 and 0.695 at 3 and 5 years. Likewise, the prognostic significance of high-risk scores was confirmed in the external validation GSE30219 cohort, showing a substantial hazard ratio of 3.2 (95% CI 2-5.2, *p* < 0.001) and moderate discriminative ability (AUC: 0.621 at 3 year follow-up; 0.655 at 5 year follow-up). Furthermore, the diagnostic ability was acceptable in discriminating LUAD patients from healthy controls, by constructing a diagnostic model using the seven key genes from the prognostic signature, with AUCs of 0.997 in TCGA and 0.807 in an independent GSE30219 dataset.

In another study [53], four lncRNAs (SNHG3, RMST, ZNF667-AS1,and COLCA1) were demonstrated to be significantly dysregulated according to the next-generation sequencing on a cohort containing 48 renal cell carcinoma paired tumor and non-tumor tissues, and further validated by qPCR. In addition, these lncRNAs were also dysregulated in patients with early relapse compared to the late-relapsing patients. The AUC of SNHG3 was 0.7332 in tumor versus non-tumor tissue (*P* < 0.0001), and 0,7071 in patients with early relapse compared to the late-relapsing (*P* = 0.009). The model based on these four lncRNAs for prediction of early relapse after nephrectomy achieved AUC 0.9241 with sensitivity 93.75% (95% CI: 71.67% to 99.68%) and specificity 71.07% (95% CI: 63,59% to 77,55%).

Another five-lncRNA prognostic signature was established in prostate cancer from TCGA database, and the prognostic risk score formula was calculated as follows: SNHG3 × 0.672549074 + LINC00857 × 0.453738256 + LINC00908 × (-1.24500487) LINC00900 × (-0.289510446) + FENDRR × 0.204942803 [54]. Kaplan Meir analysis confirmed that the high-risk group (*n* = 246) had a worse OS compared to the low-risk group (*n* = 247) (*P* < 0.01). The receiver operating characteristic (ROC) analysis for 14-year OS yielded an AUC of 0.829, signifying a high level of prognostic accuracy for the integrated five-lncRNA signature in prostate cancer. Additionally, a C-index of 0.84 (95% CI: 0.89–0.99) further underscored the signature’s robust predictive potential. These results indicate that the five-lncRNA signature could serve as a valuable tool for predicting patient outcomes and guiding treatment decisions in prostate cancer.

A risk score formula for gastric cancer prognosis prediction was created using RNA sequencing data from the TCGA database [55]. The formula considers the expression of four signature lncRNAs and is calculated as follows: SNHG3 × 0.4163 + C21orf62-AS1 × 1.4731 + AS1MIR99AHGS × 0.2341 + LINC00843 × (− 1.0637). Low-risk patients (*n* = 143) showed significantly longer RFS compared to high-risk patients (*n* = 144) (HR = 2.329, 95% CI:1.363–3.979, *P* = 0.001448). The four-lncRNA signature risk score was validated in two validation GEO data sets, with statistically significant differences in RFS time between the risk groups (validation set 1: HR = 1.368, 95% CI: 1.017–1.839, *p* = 0.03733; validation set 2: HR = 1.555, 95% CI: 1.089–2.221, *P* = 0.01444). The AUC values were 0.936, 0.827, and 0.822 for the training set, validation set 1, and validation set 2, respectively.

A prognostic model using eight glycolysis-related lncRNAs was developed for hepatocellular carcinoma using LASSO regression analysis in the TCGA database [56]. Risk scores were calculated with the formula: SNHG3 × 0.299 + SNHG12 × 0.475 + WAC-AS1 × 0.722 + AC145207.5 × 0.107 + MSC-AS1 × 0.044 + PTOV1-AS1 × 0.015 + AL031985.3 × 0.202 + MIR210HG × 0.179. ROC curve analysis (AUC = 0.779), survival analysis (*P* < 0.001), independent prognostic analysis (*P* < 0.001) showed that the model has good predictive ability. The risk score can be used as an independent factor to predict outcomes of hepatocellular carcinoma patient (*P* < 0.001).

## Concluding remarks

Numerous studies have found that SNHG3 is frequently overexpressed and promotes cancer progression in multiple tumors. High SNHG3 expression level has been associated with advanced cancer stages and patient outcomes in various tumors, suggesting that SNHG3 levels could serve as a potential indicator for diagnosis or prognosis of different cancers. Nevertheless, a large number of in-house patients with RNA expression profiles and long-term follow-up data were need to verify the classification performance and survival prediction performance of the prediction models.

SNHG3 is shown to impact cancer cell behavior, such as proliferation, apoptosis, invasion, and angiogenesis, by influencing regulatory networks in cancer through interacting with RBPs or functioning as a ceRNA, and regulating protein levels. Targeting SNHG3 holds potential for cancer treatment. To date, several strategies have been developed to target lncRNAs, and approaches directed against SNHG3 are currently under development. (1) Small interfering RNA (siRNA): siRNA binds to lncRNAs via base complementarity, guiding the RNA-induced silencing complex (RISC) to recognize and degrade the target transcript [57]. SiRNA-mediated knockdown of SNHG3 has been shown to affect various cellular functions in multiple tumor types [8, 24, 25]. (2) Antisense oligonucleotides (ASOs): ASOs are designed to be complementary to specific lncRNAs and induce their degradation through RNase H-mediated cleavage [58]. SNHG3 knockdown by ASOs has been demonstrated to inhibit migration and invasion in HeLa cervical cancer cells [59]. (3) CRISPR-Cas9 genome editing: CRISPR-Cas9 technology can delete genomes at precise locations with a specific size and high fidelity [60]. Knockout of SNHG3 expression using CRISPR-Cas9 has been shown to suppress proliferation, migration, invasion, and angiogenesis in bladder cancer cells in vitro [7]. (4) Indirect regulators: targeting upstream regulatory factors provides an alternative means to modulate lncRNA expression. STAT3 has been shown to regulate SNHG3 expression in breast cancer cells, positioning it as a potential therapeutic target for inhibiting SNHG3. Strategies targeting STAT3 can be divided into direct and indirect approaches [61]. Direct strategies involve the use of peptides, small molecules (e.g., BBI608), or oligonucleotides (e.g., AZD9150) to inhibit STAT3 phosphorylation, dimerization, or promote its degradation [62, 63]. Indirect strategies focus on upstream pathway components such as JAK or IL-6 [64]. Although druggability of STAT3 remains a challenge, emerging approaches such as PROTAC degraders (e.g., SD-36) and drug repositioning offer promising avenues [65]. Nevertheless, research on SNHG3 is still in its early stages and presents several challenges. Firstly, elucidating the detailed molecular mechanisms and regulatory networks involving SNHG3. Secondly, validating findings from cell lines and tissue studies in clinical cohorts. Lastly, developing effective in vivo delivery systems for SNHG3-targeting therapies, as current approaches (e.g., siRNA-loaded lentiviruses or plasmids) are limited to in vitro models. Additional research is required to translate fundamental SNHG3 findings into clinical applications, ultimately improving patient outcomes.

In summary, this review offers a comprehensive overview of the current understanding of SNHG3 in human cancers and aims to provide new insights and perspectives to facilitate further advancements in this field of research.

## Data Availability

No datasets were generated or analysed during the current study.
